# Three successful pregnancies in a patient with glycogen storage disease type 0

**DOI:** 10.1002/jmd2.12178

**Published:** 2020-10-26

**Authors:** Sarah C. Grünert, Stefanie Rosenbaum‐Fabian, Luciana Hannibal, Anke Schumann, Ute Spiekerkötter

**Affiliations:** ^1^ Department of General Paediatrics, Adolescent Medicine and Neonatology Medical Centre‐University of Freiburg, Faculty of Medicine Freiburg Germany

**Keywords:** glycogen storage disease, glycogen synthase, glycogen synthetase, GYS2, ketotic hypoglycemia, pregnancy

## Abstract

Glycogen storage disease type 0 (GSD 0) is a rare inborn error of metabolism due to deficiency of the enzyme glycogen synthase (EC 2.4.1.11). The disorder is clinically characterized by ketotic fasting hypoglycemia in combination with postprandial hyperglycemia and hyperlactatemia. So far, only one pregnancy has been described in a woman with GSD 0. We report a 32‐year‐old GSD 0 patient with three successful pregnancies. The diagnosis of GSD 0 was made in early childhood due to characteristic symptoms. The patient had two healthy children at the time of her first visit in our metabolic center. The diet was optimized prior to her third pregnancy with a protein‐rich diet including cornstarch and protein supplements. Pregnancy was confirmed at week 6 of gestation. Dietary management was difficult during pregnancy, especially in the first trimester due to severe nausea. Labor was induced at 37 weeks of gestation due to cholestasis of pregnancy, and the patient delivered a healthy baby girl. Perinatally, the mother received a high glucose infusion to stabilize blood glucose levels. The neonate also required a glucose infusion postnatally because of impaired glucose homeostasis. Similar to diabetic fetopathy, recurrent maternal hyperglycemia may result in hyperinsulinism of the child and trigger neonatal hypoglycemia. All four pregnancies in women with GSD 0 described to date occurred with minor complications and resulted in healthy offspring, which underpins the good prognosis and rather benign character of this rare metabolic disease. Careful monitoring during pregnancy and delivery is, however, necessary to minimize the risk of recurrent hypoglycemia for both mother and child.

**Synopsis**

Successful pregnancy is possible in glycogen storage disease type 0, but careful monitoring during pregnancy and delivery is needed to minimize the risk of recurrent hypoglycemia for both mother and child.

**Abbreviations**
GSD 0: gycogen storage disease type 0
PIGF: placental growth factor
sFlt‐1: soluble Fms‐like thyrosinkinase‐1

## 1 BACKGROUND

**FIGURE 1:**
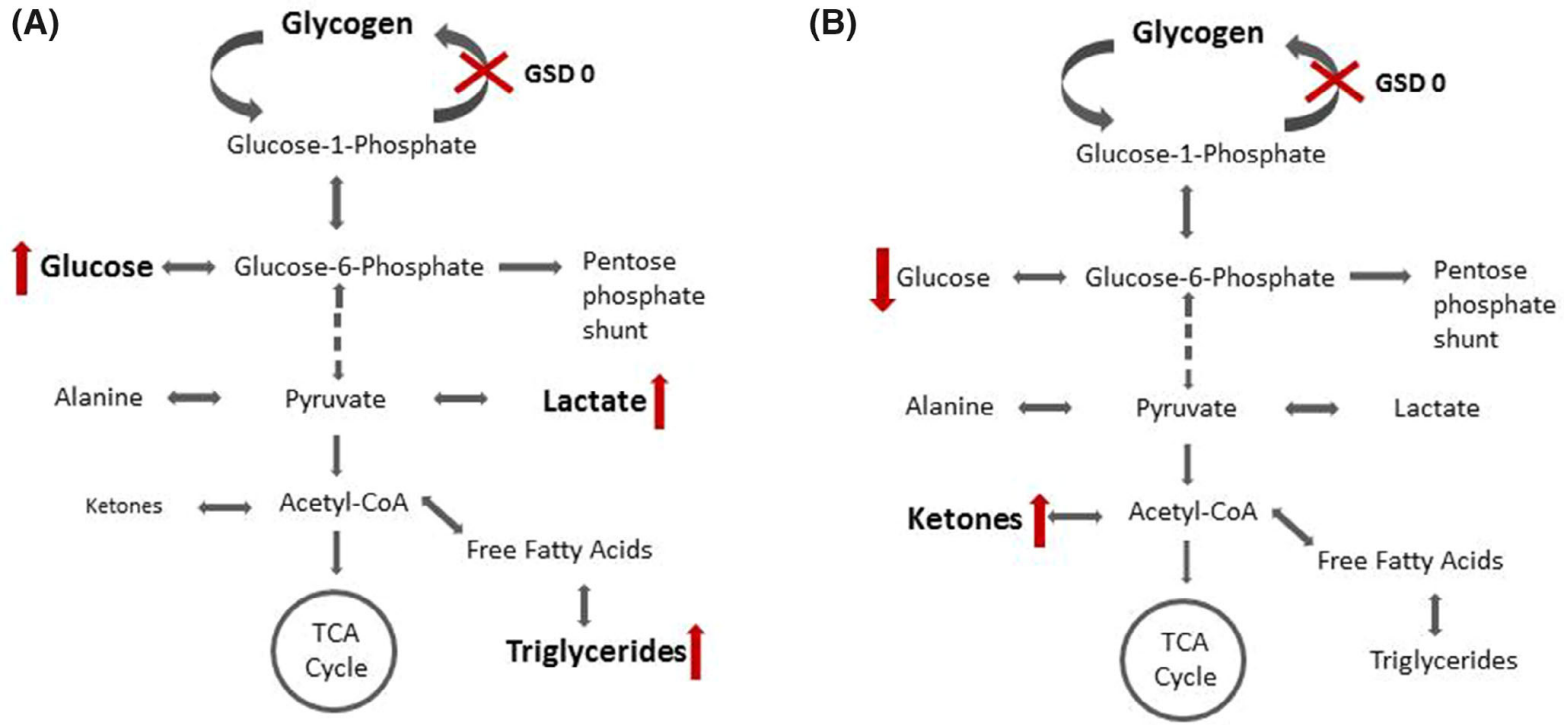
Biochemical pathways affected in glycogen storage disease type 0 highlighting biochemical abnormalities. A, Metabolic status in the fed state. In the presence of adequate glucose supply energy is derived primarily via glycolysis. A surplus intake of glucose in GSD 0 patients leads to elevated lactate and triglycerides. B, Metabolic status in the fasted state. Due to the lack of glycogen biosynthesis, an insufficient supply of glucose in GSD 0 patients leads to the upregulation of ketone body production to sustain energy demands. The inability to store glucose units as glycogen requires tight control of glucose intake in GSD 0 patients

Glycogen synthase deficiency (OMIM #240600), also known as glycogenosis (GSD) type 0, is a rare inborn error of glycogen metabolism due to mutations in *GYS2*.^1^ Although the disease was described in 1963,^2^ only about 40 cases of GSD 0 have been reported in the literature so far.^3^ The disorder is clinically characterized by ketotic fasting hypoglycemia in combination with postprandial hyperglycemia and hyperlactatemia.^4^ An overview on the pathophysiology and biochemical abnormalities in the fasting and postprandial state is given in Figure 1. Due to the inability of patients to store glucose as glycogen in the liver, hepatomegaly is no typical clinical feature of GSD 0, although mild hepatomegaly may appear from a fatty liver.^5^ Further clinical symptoms comprise lethargy, morning drowsiness, pallor, nausea, vomiting, and seizures following overnight fasting. Growth failure is also common with both short stature and failure to thrive.^5^ The prognosis of GSD 0 seems to be excellent, and long‐term complications have not been described to date.^4^ First symptoms are usually observed in late infancy or early childhood. There is no clear genotype‐phenotype correlation in GSD 0.^3^

The main treatment goal is the prevention of hypoglycemia and to minimize the systemic acidosis by preventing postprandial lactic acidosis and fasting ketosis.^4^, ^6^ As patients with GSD 0 are able to produce glucose from protein via gluconeogenesis, dietary treatment is based on a protein‐rich diet with complex carbohydrates. Some patients may require supplementation with uncooked cornstarch to maintain normoglycemia, even on a high protein diet.

There are few reports of adults with GSD 0, and the oldest documented patient is 34 years old.^1^ To our knowledge, only one pregnancy has been reported in a woman with GSD 0.^7^ A 26‐year‐old patient delivered a healthy term girl, but overnight hypoglycemia and ketonemia were observed during the second and third trimester.^7^

We here describe a 32‐year‐old patient with three successful pregnancies. The management and challenges during gestation and delivery are discussed.

## 2 CASE PRESENTATION

The patient is a 32‐year‐old woman who was diagnosed with GSD 0 at the age of 4 years. A clinical description until age 8 years has been published previously.^8^ She developed normally until the age of 3.5 years when she was noted to be drowsy in the mornings and occasionally vomited. Laboratory testing revealed hypoglycemia with marked ketonuria. Liver biopsy performed at 4 years yielded a reduced glycogen content of 0.9 g/100 g (normal 2.4‐6.4 g/100 g) and a very low glycogen synthase activity. A high protein diet with frequent daytime meals, a late dinner, and two doses of 30 g of uncooked cornstarch during the night were recommended. On this regimen, the patient showed normal growth and development, her BMI at the age of 5 years was normal with 16.1 kg/m^2^. Further hypoglycemias only occurred during physical activity, such as swimming or other sports, and regular blood glucose monitoring was abandoned. Mutation analysis in *GYS2* was performed and yielded compound heterozygosity for the two novel variants, c.1015G > C (p.A339P) and c.1472T>G (p.M491R). From the teenage years until the age of 27 the patient was not regularly followed and did not take cornstarch supplements. At the age of 27 she presented to the metabolic center of her region while planning her first pregnancy. At age of 28, she gave birth to a healthy girl (birth weight 3610 g, 73th centile, 39 + 1 weeks of gestation), and 1.5 years later a healthy boy was born (4195 g, 94th centile, 39 + 1 weeks of gestation, large for gestational age). The first pregnancy was complicated by severe nausea and vomiting, and the patient was admitted to the hospital twice for glucose infusions due to recurrent hypoglycemia. During the second pregnancy the patient suffered from dizziness, but was otherwise well. Both pregnancies and deliveries were managed without major complications, and both children show normal growth and development so far.

**FIGURE 2:**
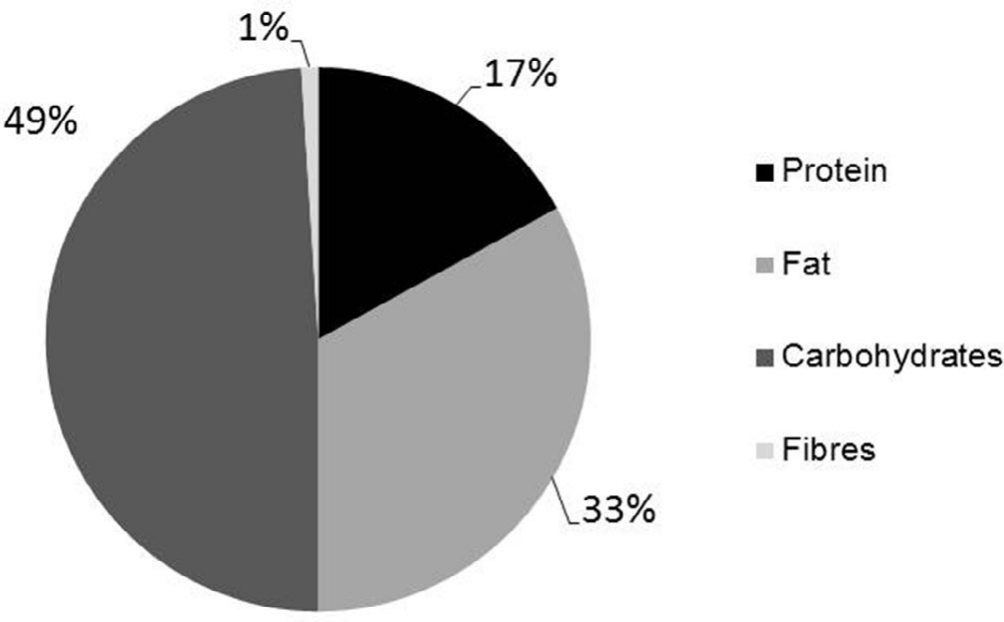
Composition of the diet at first presentation. Protein intake was low with only 0.9 g/kg/day (recommendation for GSD 0 patients >2 g/kg/day)

The patient first presented at our metabolic clinic at the age of 31 years. At that time, she did not follow a specific diet, and blood glucose levels were not regularly monitored. A three‐day dietary protocol revealed a slightly hypocaloric diet (1700 kCal/d, normal for age 2200 kCal/d) with a protein intake of only 0.9 g/kg/d accounting for 17% of the daily energy intake (Figure 2). The patient reported to feel unwell during both hypoglycemic and hyperglycemic episodes. Muscle pain and muscle weakness were not reported, but she complained about fatigue. Blood glucose monitoring for 2 days revealed no relevant hypoglycemias (<3.9 mmol/L). Laboratory testing yielded normal transaminase and creatine kinase acitivites. Abdominal ultrasound was also normal.

Continuous glucose monitoring and intermittent measurements of blood ketones were initiated. A protein‐rich diet (about 3 g of protein/kg body weight/day) with protein supplements, complex carbohydrates, and the reduction of simple sugars was recommended. Sixty grams of Glycosade were given at bedtime. Under this regimen blood glucose levels stabilized and hypoglycemia below 3.3 mmol/L occurred rarely. Total protein concentration was within the normal range (7.2 g/L).

The patient became pregnant for the third time and contacted us at week 6 of gestation. Continuous glucose monitoring was performed throughout pregnancy, and the diet was adapted accordingly. An overview on the supplementation of Glycosade and protein is given in Table 1. During the first trimester the patient suffered from severe nausea and had difficulties to take the protein supplement. The metabolic situation was unstable, especially toward the end of the first trimester, with a very short fasting tolerance between 1 and 2 hours during day and night. Hyperglycemic episodes with glucose levels above 8.3 mmoL/L became more common. In the second trimester, nausea subsided, and the metabolic situation stabilized. We recommended to not perform the oral glucose tolerance test that is routinely done in week 24 of gestation. During the third trimester the patient experienced a strong tendency toward hyperglycemia although the nutritional intake remained unchanged. Fetal growth was normal throughout pregnancy. The total weight gain during pregnancy was 17 kg, thereof 5 to 6 kg within the first trimester.

**TABLE 1:** Overview on body weight, Glycosade and protein supplementation, and metabolic stability throughout pregnancy

Point of time	Glycosade®	Protein supplement	Fasting tolerance/metabolic stability
Before pregnancy	60 g at bedtime	30 g at bedtime	Low frequency of hypoglycemias
First trimester	60 g at bedtime	Supplementation difficult due to severe nausea	Very unstable metabolic situation with a fasting tolerance of maximum 2 hours at the end of the first trimester
Second trimester	60 g at bedtime 30 g at 3 am	1 to 2 doses of protein (30 g) throughout the day, 30 g at bedtime	Metabolic situation stabilized, fasting tolerance 3 hours during the day, 5 hours at night
Third trimester	60 g at bedtime 30 g at 3 am	No protein supplementation from weeks 24 to 30, later 30 g at bedtime and dditional 1 to 2 doses throughout the day	Tendency toward hyperglycemias, fasting tolerance 3 hours during the day, 5 hours at night

At 35 weeks gestation the patient complained about progressive pruritus. Laboratory testing revealed elevated transaminase activities (GOT 129 U/L, GPT 225 U/L, normal 10–35 U/L), alkaline phosphatase (182 U/L, normal 35‐105 U/L) and bile acids (26.5 μmol/L, normal <10 μmol/L). The concentration of gamma GT was normal (17 U/L, normal <40 U/L), and no proteinuria was present. Cholestasis of pregnancy was diagnosed and treatment with ursodeoxycholic acid was initiated. Preeclampsia was ruled out (sFlt‐1/PIGF ratio 5, normal <38). Labor was induced at 37 weeks gestation. During delivery the patient received a high glucose infusion with 10 g glucose/h, under which blood glucose levels remained stable within the normal range. She delivered a healthy girl by vacuum extraction due to fetal bradycardia. The Apgar score was 7/4/7, cord blood pH 7.09 and the base excess −6 mmol/L. Her birth weight was 3640 g (75th centile). The child required non‐invasive mechanical ventilation for 6 days due to respiratory distress. No laboratory signs of infection were observed. Glucose infusion was necessary until day 5 due to low blood glucose concentrations. Echocardiography of the neonate on day 2 showed a persistent arterial duct that was no longer detectable on day 7. The myocardium was slightly thickened which was considered to be due to diabetic fetopathy. Ultrasound of the brain was normal.

Maternal blood glucose concentrations postpartum were more stable than during the last trimester, and no hypoglycemias or severe hyperglycemias occurred during early lactation. One dose of Glycosade (60 g) together with protein powder was sufficient to maintain normal glucose levels overnight. The child was mainly breastfed with supplementation of formula milk.

## 3 DISCUSSION

Since GSD 0 is a rather benign disorder with an excellent prognosis more and more patients will reach child‐bearing age. Nonetheless, pregnancy in patients with GSDs poses unique challenges during gestation and delivery. Due to the metabolic demands of the fetus and hormonal adaptations during pregnancy,^9^ women with GSD 0 are prone to metabolic derangements with hypoglycemia, hyperketonemia, and hyperlactatemia. Therefore, careful monitoring is necessary throughout pregnancy, and maternal blood glucose levels need to be maintained in a healthy range for the safety and proper development of the fetus.^9^ For other types of GSDs, it has been shown that good metabolic control before conception and throughout pregnancy is directly related to successful outcomes.^9^

We report the second GSD 0 patient with successful pregnancies. The first patient described by Byrne et al was followed from week 18 of gestation onwards.^7^ She remained well in pregnancy on a high protein diet, and no further dietary adaptations were applied. However, overnight hypoglycemia and ketonemia were observed during the second and third trimesters. Our patient suffered from severe nausea during the first trimester, and the fasting tolerance was very short, even with supplementation of Glycosade. Severe hypoglycemias could however be prevented with dietary adjustments; intermittent ketone measurements in the mornings showed ketone levels <0.2 mmol/L. During the second trimester the patient remained well with good metabolic stability. As the patient did not perceive a clear benefit of protein supplementation, the intake was discontinued between weeks 24 and 30. In Germany, an oral glucose tolerance test is performed in the routine care of pregnant women in week 24 of gestation to screen for gestational diabetes. Because a high glucose load may result in severe hyperglycemia and lactic acidosis in a patient with GSD 0, we recommended to not perform this test in our patient.

Ketone levels were checked routinely in our patient throughout pregnancy, and were always low (<0.5 mmol/L). Pregnant women usually have two to four times higher ketone concentrations after an overnight fast compared to non‐pregnant women.^10^ In the GSD 0 patient reported by Byrne et al ketone levels were even 10 times higher than those seen in non‐pregnant women.^7^ A study that investigated correlations between antepartum maternal metabolism and intelligence in the offspring found significant correlation between intelligence in early childhood and maternal plasma hydroxybutyrate levels in the third trimester of pregnancy.^11^ Interestingly, no correlation was found between maternal hypoglycemia in pregnancy and the intelligence of the offspring in early childhood.^11^ If the cholestasis of pregnancy observed in our patient is associated with the underlying metabolic defect, remains unclear, and it is more likely that it was coincidental.

Good planning and interdisciplinary collaboration between metabolic physicians and gynecologists is necessary to guarantee a safe setting during delivery for patients with GSDs. Administration of a high glucose infusion and regular monitoring of blood glucose, ketones, and blood gases is necessary during labor.

Apart from respiratory distress, the newborn of our GSD 0 patient also showed a tendency toward hypoglycemia, which is reminiscent to that seen in newborns from mothers with gestational diabetes. In healthy pregnant women hormonal changes during pregnancy with a rise in anti‐insulinergic hormones, such as human placental lactogen, progesterone, and oestrogen, lead to increasing insulin resistance.^9^ During a normal pregnancy there is a doubling of insulin secretion from the end of the first to the third trimester.^7^, ^12^ The blood glucose profile of a GSD mother may resemble that of a diabetic mother with frequent postprandial hyperglycemias. The β cells of the fetal pancreas are able to secrete insulin from week 12.^13^ If the pregnant woman is hyperglycemic, glucose passes across the placenta resulting in an increased fetal blood glucose concentration. If maternal hyperglycemia occurs often, this may lead to hyperinsulinism in the child, and it is well conceivable that this may result in an impaired glucose homeostasis during the neonatal period. In favor of this theory, the offspring showed a slightly thickened myocardium as seen in newborns with diabetic fetopathy. Additionally, all three children of the patient had a birth weight above the 70th centile, and in the second pregnancy, labor was induced due to suggested fetal macrosomia. Interestingly, Byrne et al. have studied glucose, insulin and C‐peptide levels in their patient before pregnancy and at 24 and 32 weeks gestation and could not find higher levels in pregnancy compared to pre‐pregnancy values,^7^ which might have been due to an inadequate energy supply with frequent hypoglycemias and ketonemia in this woman.

Although genetic testing has become widely available for patients with unclear diagnosis, the number of GSD 0 patients reported in the literature is still very low. It has been hypothesized that this disease may be underdiagnosed, since asymptomatic siblings have been identified in several GSD type 0 families.^8^, ^14^ GSD 0 can, for several years, remain silent or may take an oligosymptomatic and mild course^8^, ^15^, ^16^ as was the case in our patient who showed first symptoms only at the age of 3.5 years. Few adolescent and adult patients with GSD 0 have been reported so far, and their clinical course suggests that the fasting tolerance increases with age.^7^, ^15^ This was also observed in our patient who presented with hypoglycemia and hyperketonemia in early childhood, but remained well since the age of 8 onwards, as shown by her requiring minimal to no medical follow‐up. Organ‐specific long‐term complications as seen in other GSDs, such as hepatic adenomas, cirrhosis, kidney dysfunction, and muscular abnormalities, have not been reported in adolescents or adults with GSD 0.^4^ Family planning and starting a family life are important components of adult life. The fact that all four pregnancies in the two women with GSD 0 described to date were successful and without major complications, suggests that patients with GSD 0 are not limited in this respect by their medical condition. As optimal metabolic control should ideally be reached before conception, regular follow‐up in GSD 0 patients should not be discontinued during teenage years, even in patients without medical problems.

## CONFLICT OF INTEREST

The authors declare that they have no competing interests.

## AUTHOR CONTRIBUTIONS

SCG was responsible for clinical management of the patient and drafted the manuscript including Figures 1 and 2. SR‐F was responsible for the dietary treatment as nutrition expert. LH, AS and US were involved in the clinical care and laboratory work‐up of the patient. All authors edited and proofread the manuscript prior to submission.

## CONSENT FOR PUBLICATION

The patient gave her written informed consent for the publication of this case report.

## AVAILABILITY OF DATA AND MATERIALS

Not applicable.
